# Supplementary material for: The causal relationship between the human gut microbiota and pyogenic arthritis: a Mendelian randomization study
Source: Front Cell Infect Microbiol. 2024 Nov 26;14:1452480. doi: 10.3389/fcimb.2024.1452480 (PMC11629706; doi:10.3389/fcimb.2024.1452480)
Supplement: Supplementary file 1 [file DataSheet1.zip › mendelian test/ebi-a-GCST90027474.csv_leave_one_out.pdf]

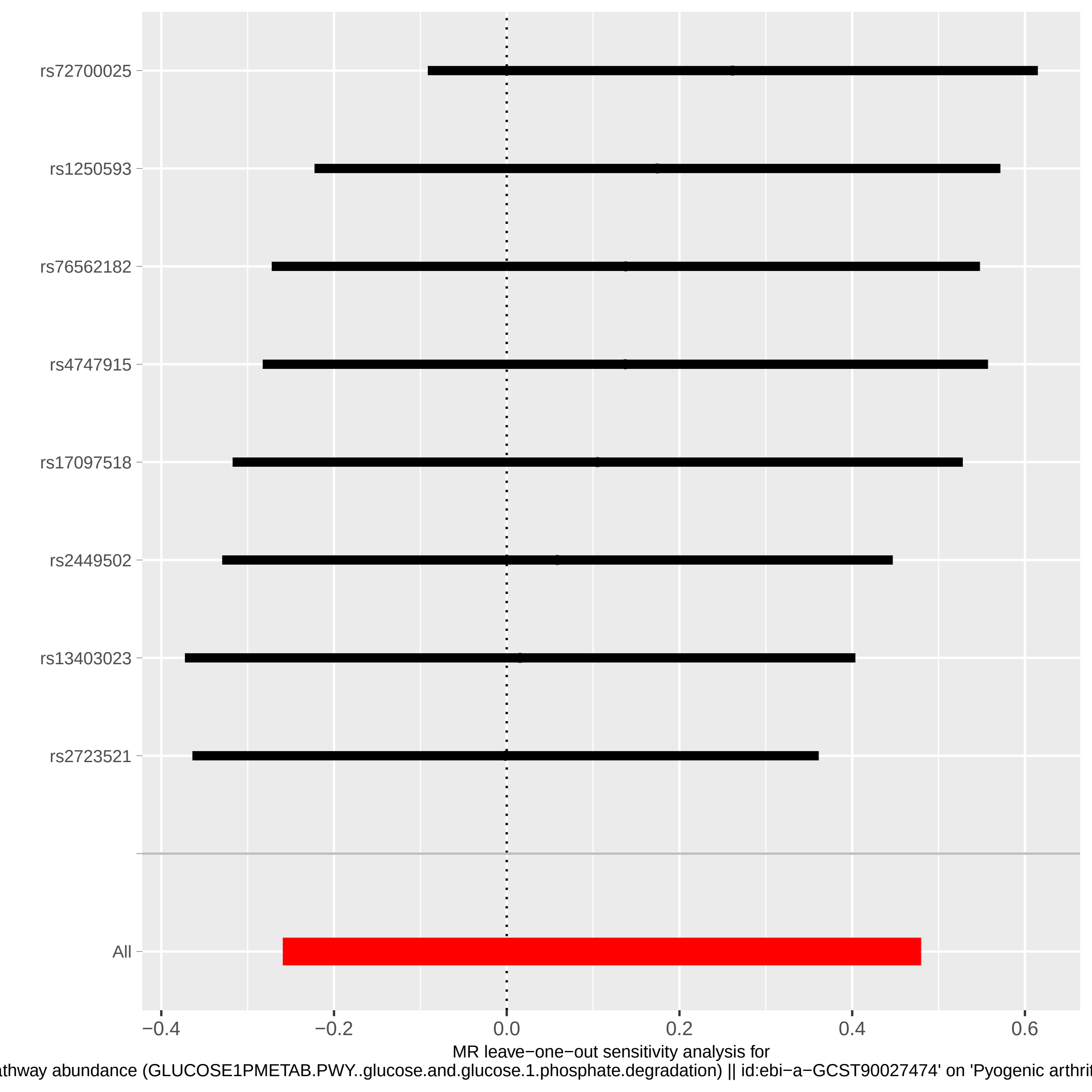
rs72700025
rs1250593
rs76562182
rs4747915
rs17097518
rs2449502
rs13403023
rs2723521
All
−0.4
−0.2
0.0
0.2
0.4
0.6
MR leave−one−out sensitivity analysis for
athway abundance (GLUCOSE1PMETAB.PWY..glucose.and.glucose.1.phosphate.degradation) || id:ebi−a−GCST90027474' on 'Pyogenic arthrit
